# Using Eye Movements to Understand how Security Screeners Search for Threats in X-Ray Baggage

**DOI:** 10.3390/vision3020024

**Published:** 2019-06-04

**Authors:** Nick Donnelly, Alex Muhl-Richardson, Hayward J. Godwin, Kyle R. Cave

**Affiliations:** 1 Department of Psychology, Liverpool Hope University, Liverpool L16 9JD, UK; 2 Department of Psychology, University of Cambridge, Cambridge CB2 3EB, UK; 3 Psychology, University of Southampton, Southampton SO17 1BJ, UK; 4 Department of Psychological and Brain Sciences, University of Massachusetts Amherst, Amherst, MA 01003, USA

**Keywords:** visual search, eye movements, X-ray images, security screening

## Abstract

There has been an increasing drive to understand failures in searches for weapons and explosives in X-ray baggage screening. Tracking eye movements during the search has produced new insights into the guidance of attention during the search, and the identification of targets once they are fixated. Here, we review the eye-movement literature that has emerged on this front over the last fifteen years, including a discussion of the problems that real-world searchers face when trying to detect targets that could do serious harm to people and infrastructure.

## 1. Introduction

The job of an airport screener is to stop a wide range of prohibited items being taken on board aircraft. Some of these prohibited items are threats (e.g., guns, knives, explosives) and some are restricted for other reasons (e.g., liquids, narcotics, etc.). Airport screeners search for the presence of these prohibited items in two or three-dimensional images derived from X-raying screening trays. Their job is, therefore, one that requires the inspection of visual images for the presence of prohibited items.

While the ability to detect prohibited items in X-ray images of baggage has been explored for some decades, it came on to the radar of experimental psychologists following the terrorist attacks on the World Trade Center in New York in 2001. From this time onwards, researchers sought to better understand the perceptual and cognitive challenges associated with airport baggage screening. In this review, we report some key data from experimental studies in which eye movements have been recorded in order to better understand how, when, and why errors are made during the search. The results have been informative from both a theoretical and practical perspective.

## 2. What Are the Images Used in Baggage Screening?

Compared to most real-world search stimuli and medical images, baggage images have less structure. The contents and arrangement of items in bags and of bags on screening trays are difficult to predict. This leaves the screener with few expectations about where these objects should appear within the image, and thus makes decision making more uncertain than in most other search tasks. 

**Figure 1:**
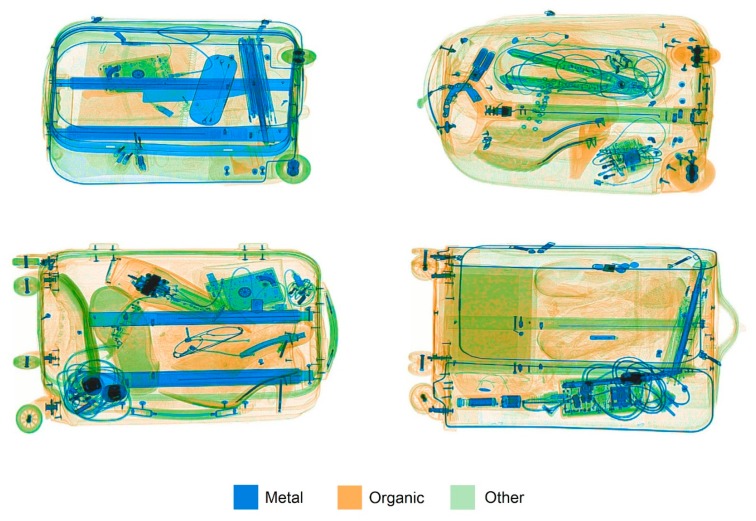
Example X-ray images of bags, two contain threats (top left—knife; bottom right–pistol); images from the CaSePIX image library [1]. Reproduced with permission from the copyright holder Dr Greg Davis.

Baggage images are typically, but not always, colored (or more correctly *pseudo-colored*) using a standard color mapping (see Figure 1). The artificial color mapping depicts the density of the objects in the image, and also provides some information about object materials. Interpreting density is relatively straightforward: areas with no material or with very low density appear as white, with higher density regions appearing as darker and more saturated colors. If a region is so dense that no X-rays pass through it, it is depicted as black. Interpreting X-ray images is tricky because these arbitrary color mappings produce a visual world that is very different from what we are accustomed to.

The depiction of information about the materials making up the objects is more complex. The X-rays that have passed through the objects can be used to infer an atomic number, which is then used to put each part of the image into one of three categories. Those regions with the lowest atomic numbers will include most explosives, but will of course include many non-threatening organic materials as well, such as foods and fabrics. These regions of the image are assigned an orange hue in the standard color mapping. Regions with the highest atomic numbers include metal weapons such as guns and knives, but also many harmless objects, and appear as blue. Atomic numbers in between these ranges are often shown as green, and can generally be regarded as less threatening because they are not metal and are not explosives.

The challenge to the interpretation of X-ray images is increased because all but the densest objects have a certain amount of transparency. Once X-rays have been emitted from the X-ray sources and passed through one object, they may pass through other objects before striking the collector and being registered. Thus, one part of the image often depicts information about multiple objects. This overlap introduces the additional problem of segmenting the different objects from one another. 

There is another aspect of object overlap in X-ray images that is very different from our usual experience. When an orange, green, or blue hue is assigned to each part of the image, it is based on an *average* of the atomic numbers of the different materials that the X-ray beam has passed through. Thus, if an organic object with atomic number in the low range overlaps with a metal object with atomic number in the high range, the region of overlap in the image may be depicted as belonging to the middle range. 

Clearly, interpreting overlapping objects in X-ray images requires careful consideration. Adding to the challenge is that these searches are often conducted in a busy work environment with time pressure from long lines of passengers who are concerned about missing a flight. Thus, it is important for us to find ways to make the task easier and to increase accuracy and efficiency. This is particularly important in light of the fact that, although search times (and limits) vary from airport to airport and from country to country, screeners often will take around only six seconds to complete their searches [2].

Recent innovations to security screening include dual view where screeners make their decisions from interpreting two orthogonal views (and sometimes a photographic image of the tray as well) [3]. Computed tomography (CT) scanners have also become available as an alternative to more traditional X-ray machines. An apparent advantage of CT scanners is that they can generate a 3D image of a tray which can be manipulated on-screen, including advancing through 2D slices of the full 3D volume [4]. The challenge to threat detection in baggage search that these conditions create is altered but not fundamentally changed by recent innovations aimed at supporting image interpretation by providing richer imagery.

While these advanced imaging systems add information that should help in the detection of prohibited items, they may also create new challenges. We note two of these challenges here as we think they are important, despite being largely unexplored. First, search and interpretation from viewing two orthogonal views requires the ability to make eye movements to the same or related locations on the different images showing orthogonal views. As far as we are aware there is only a single unpublished study on the success and utility of making these eye movements with respect to baggage screening [5]. Second, radiological imaging requires searching for targets in slices of a 3D volume, but what is being imaged in medical imaging is of a known structure and complexity, and what is being identified are variations from a norm. The risk to baggage screening of using this technology is that features consistent with an early emerging target may be ‘tracked’ through slices, leading to misses from targets appearing later in the sequence of slices. We know of only a single study that has explored an analogue of this situation [6].

## 3. What Are the Decisions Made by Baggage Screeners? 

While we refer to baggage search throughout this review, in line with the existing literature on this topic, in the real world of baggage screening, the images are more correctly defined as the contents of *screening trays*. Screening trays can contain bags, laptops, and other items that are routinely X-rayed such as keys, wallets and mobile telephones. The clarification is an important one to make. It is important to be clear that the visual search and threat identification challenge that these different situations deliver for screeners changes somewhat unpredictably on a tray-by-tray basis. The important point is that some decisions can be made without the need for extensive searching at all, but on the basis that the gist of the tray indicates the presence of very little or nothing that comes close to resembling a target. We define gist here in a very limited way as providing an estimation of the clutter of the objects within a tray. Virtually empty trays may allow rejection on the basis that the limited material present can easily be discounted as resembling a target. We do not mean to imply gist emerging from object co-occurrence, configurations or scene backgrounds [7].

A screener can either “clear” a bag and let it pass, or “reject” it. A bag may be rejected because a prohibited object(s) can be seen and readily identified in an image. However, a bag may also be “rejected” simply because the screener cannot be certain that prohibited objects(s) are not present in an image, probably because there are many objects overlapping one another. In either case, the decision to reject bags usually leads to further investigation through hand searching. 

## 4. The Security Search Task

Having considered the images from which ‘clear’ and ‘reject’ decisions are made and the nature of those decisions, we now turn to eye movements and the security search task. The color coding of atomic density allied to the uncertainties created by contents, the arrangement of contents, and viewpoint mean that color is often used to guide attention to possible targets. Fortunately, we know quite a lot about how people search for color and the eye movement behavior associated with this search. In addition, studies have explored the costs associated with simultaneously searching for more than one color, as is the case when searching for, for example, the orange indicating possible plastic explosive and the blue/black indicating possible wire, guns or knives.

There are a number of prominent models of visual search [8,9,10], and fewer such models of visual search and eye movement behavior [11,12]. Despite their differences, the models of search share the common idea that, during the search, our limited resources need to be directed to given objects in order to determine whether those objects are targets [13]. In the classic Guided Search model, which has been revised several times since its inception [8,14,15,16], attention is directed towards target-similar objects, one at a time, until a target is found, or until the searcher decides to quit. The decision to terminate search can depend on a number of factors, including the time spent searching and errors made on previous trials [17]. Models of eye movements and visual search share much the same overall architecture, though they do generally focus more on the mechanisms that govern when and where to move the eyes during a search [11,18,19].

The recording of eye movements during the search opened up new avenues for understanding the information processing that takes place during the search. Many early studies of eye movements and the search of complex images involved radiographic image screening. These studies adapted and developed the language of guidance in visual search to focus on the fractionation of what takes place in terms of information-processing during visual search [20,21]. Put simply, by recording eye movement behavior, researchers were able to determine when, how and why targets were missed; this was, and still is, a question of vital importance when it comes to real-world search tasks in which missing a target can have severe consequences. These studies developed a new framework for understanding eye movements during the search by focusing on failures of *guidance* and failures of *decision-making*.

Failures of guidance were measured using three eye movement metrics: namely, the probability that a target was fixated during a search, the speed at which a target was fixated during the search, and the number of nontargets fixated. Connecting with classic models of search, such as Guided Search [8], under this view, an easier search task can be conceptualised as one wherein the target is directly and rapidly fixated. As a search task becomes more difficult, there is a longer delay before the target is fixated, as more and more nontargets are fixated, and the target is less likely to be fixated at all. As a direct consequence of this reduction in the efficacy of guidance, targets in more difficult search tasks can then be missed purely because of a failure in guidance. An early eye tracking study by McCarley and colleagues [22] found that after practice, subjects fixated fewer nontargets, but were no more likely to fixate the target. We will discuss what factors make search tasks more difficult in the context of baggage screening in more detail below.

Even if a target is directly fixated, this is not a guarantee that it will be detected. Such failures of decision-making in search can also be measured using two eye movement metrics: the time between fixating the target and identifying it as a target, and the probability of detecting a target after fixating it. When a target is more complex or difficult to recognise, this increases the time required to identify it, and, worse still, reduces the probability that it will be detected even after being directly fixated. The study mentioned earlier by McCarley et al. [22] found that search practice substantially improved target identification, although some of that improvement was tied to specific target images.

With this basic framework now set out, we will turn to outlining several ways in which exactly these forms of error have been studied extensively in the context of tasks inspired by airport X-ray baggage screening, beginning with the study of target templates in baggage search.

An understanding of the time taken to search baggage comes from studies of attentional control and guidance. A number of different theories of attentional control [8,10] are built on the assumption that early visual processing provides limited types of information that can be used to guide attentional selection. In difficult searches like baggage search, this attentional guidance is based on a mental representation with information about the target to be found, sometimes called the “target template”. Once attentional guidance has determined which portions of the input should be selected, those portions are then processed more fully to recognize objects and interpret their configurations relative to one another. In general, the information stored in the target template seems to be constrained by the rather severe limitations that apply to visual working memory. Despite those limits, visual search can be very flexible, as demonstrated by experiments by Wolfe and colleagues [23,24] in which participants are asked to search for as many as 100 different targets. These searches probably do not benefit from the accurate guidance that is possible when searching for a single color or orientation.

In bags that are largely empty, the guidance of attention may be so precise that attention is quickly directed to the search target, or the absence of any target can be quickly determined. Guidance may instead be imprecise, perhaps because it has not been possible to develop an effective target template (although even target templates that inaccurately specify target features afford guidance proportional to their target similarity [25]). In addition, it may also be the case that a bag is so densely packed with multiple metal and organic objects that focused attention is required to segment and identify and possible objects. Many baggage searches will fall somewhere in between these easy and difficult extremes. In general, attention should be guided toward the dark blue and black regions that might be metal weapons, and to the orange regions that might be explosives. By preventing attention to the light and green regions, guidance can limit the amount of time spent in attentional processing.

Attentional guidance is fairly accurate when a target is known to have a specific color [26]. Information about the target color can be used to form a mental representation or template to guide attention toward items with similar colors, avoiding most of the other colors. Security search is somewhat more complicated than this simple single-color search, however: the threat targets can be either black, blue, or orange, and the regions that can be ruled out based on color can be either green or low-saturation colors close to white. Performance in searches for multiple targets is often worse in the search for two targets than in the search for a single target, although the nature of the cost varies depending on the task. Sometimes, accuracy is lower or response times are longer in dual-target search, as shown by Menneer and colleagues [27] in searches for abstract target stimuli defined by color, orientation, or shape. Menneer et al. [28] also found dual-target costs in accuracy for searches among X-ray images of objects, as long as the two targets differed from one another in color. Eye-tracking studies, both with abstract shapes [29] and with X-ray images [26] have helped to illustrate one source of the dual-target cost. In those experiments, participants who were searching for two types of targets with different colors made a number of fixations to distractors that had colors different from either target.

The drop in search efficiency between 1-color search and 2-color search was explored with a set of very basic abstract stimuli by Stroud et al. [30]. The target was distinguished from distractors by a difficult shape discrimination, but it could nonetheless be found quickly if its color was known in advance. In 1-color search, fixation rates to colors that were very different from the target were low, demonstrating effective color guidance. However, when the target could appear in either of two very different colors, there were many more fixations to colors that were very different from the distractor. Dual-target search was much slower because participants were spending time fixating distractors that should have been excluded by color guidance. Search guidance could be degraded further by expanding the set of possible target colors to eight, so that the range of possible colors covered half of the possible hues [31]. The implication of this finding for baggage screening is clear: the simultaneous search for more than one target reduces guided eye movements to targets and increases unguided eye movements to things that are very unlikely to be targets. The time limited aspect of baggage screening makes the increased number of unguided fixations in multiple target versus single target search a significant problem when considered in the context of the job.

While color may be the best cue to guide attention in baggage search, it is not a reliable cue to the presence of prohibited items, as many non-prohibited items are also coded in orange and blue. Participants in these studies have the option to employ color guidance or not in these particular tasks because the target can often be identified by shape. The Stroud et al. study shows that if many of the items with the target color also have a shape that makes them a distractor, then participants are much less likely to guide their eye movements by color, but if most of the items with the target colors are actual targets, then there will be a stronger tendency to direct fixations to the target colors and away from colors that were never targets [31]. This pattern suggests that participants have some high-level control over how guidance is used in search: if they expect color to lead them to a target on a high proportion of trials, then they will employ color guidance more often. For baggage screeners to make use of color to guide their eye movements, they may well need to resist this tendency, and instead decide to use color guidance, despite the low likelihood that what is fixated will be a prohibited item.

Other experiments have demonstrated flexibility in search guidance that may be informative to understanding how to manage searching for multiple targets. In an eye-movement study by Beck, Hollingworth, and Luck [32], participants could either search for two target colors simultaneously, or search first for one color and then switch to the other, depending on the instructions. When left to choose their search method on their own, participants seem to do something in between purely simultaneous and purely successive search: they often switch between fixating one target color and fixating the other, but at a given moment during the search they seem to favour one target over the other [33]. 

In summary, when a search task, like baggage search, requires holding two colors in working memory, participants are more likely to perform the task without guiding attention by color, even though guidance may make their search more efficient. This occurs whether both colors are search targets, or one is held for another task [34]. When guidance is not used, there may be many fixations to distractors that could be avoided, and the search may take much longer to complete. 

## 5. Identification of Complex, Overlapping Transparent Objects

As noted above, one of the key problems associated with searching through baggage X-rays is the fact that they contain a wide array of varied overlapping and transparent objects. The manner in which these objects combine creates a uniquely difficult task wherein objects can be difficult to identify (see reference [35] for an excellent illustration of this point).

Applying what is known regarding human search behavior to the basic properties of the images that screeners search through is a difficult task, mainly because the vast majority of visual search experiments have utilised displays wherein objects do not overlap with one another. Studies that have examined the deleterious effect of searching through cluttered, overlapping displays [36,37] find that search is impaired for these difficult images. 

More recently, we conducted a series of experiments [38] that focused on the problem of overlap and transparency in search and also recorded the eye movement behavior of participants as they searched the displays. We found that, for opaque displays, target detection rates fell substantially and reaction times increased when object overlap increased. We also found that perceptual selection and perceptual identification errors increased substantially as overlap increased. Moving to transparent displays, the same basic pattern emerged, with the important difference that reaction times were even longer for higher levels of overlap than in the opaque displays. Crucially, this was likely to be the result of the fact that, in transparent images, the complexity of the displays *obscures* but does not *remove* information as it does in opaque displays. The cost associated with perceptual identification in transparent images led to long verification times because of the need to examine multiple different possibilities of grouping and object identity before a final decision could be reached.

Despite the apparent concerns regarding the effects of overlapping images in complex displays, there is reason to be confident that there may be ways to assist those searching these difficult images. In the same set of studies [38], we also found that presenting the objects on separate three-dimensional depth planes aids search and ameliorates the effects of overlap, suggesting that future research would benefit from further understanding the benefits of depth in aiding in the segmentation and identification of complex, overlapping objects during the search.

## 6. The Problem of Low Prevalence 

It is fortunate that targets in X-ray baggage are very rare indeed, but the relative scarcity of targets can influence the likelihood of detecting those targets once they finally do appear. One line of studies focuses on the prevalence of targets, which is defined as the proportion of trials on which a target is presented. These studies demonstrate that low target prevalence, e.g., 2%, results in a reduction in the target detection rates compared to higher prevalence levels, e.g., 50% [39,40,41].

Early studies of this prevalence effect used behavioral measures, including response times, response accuracy, and signal detection theory measures [42]. The general finding was that reductions in target prevalence resulted in a shift in the response criterion such that participant responses became more conservative and less likely to respond that a target was present. By this we mean that their hit rate and false alarm rate were reduced and target-absent responses were sped up relative to when target prevalence was higher [41,43,44,45,46,47].

Later and more recent studies of eye movement behavior in conditions of varied prevalence have helped to elaborate on how, when and why rare targets are missed when prevalence is low. For example, a reduction in target prevalence increases failures of perceptual selection, whereby searchers fixate fewer objects in each trial as prevalence is reduced [48]. It is worth noting that efforts to overcome this shortcoming by providing feedback in relation to where fixations have and have not been made are unlikely to help [49,50].

Moreover, the failure of perceptual selection is added to by the fact that targets are fixated more slowly when target prevalence is low [48]. Moreover, a reduction in target prevalence also increases the likelihood of perceptual identification errors, with participants not just being slower to identify targets after fixating them, but also less likely to detect targets after fixating them [48,51,52].

It is worth noting that some of the challenges caused by the low prevalence of actual threat items are mitigated in the real world of baggage screening by the inclusion of other prohibited items that must be searched for. Even without the inclusion of other prohibited items that must be searched for, the detection of threat items may be aided by the inclusion of Threat Image Projection (TiP) items amongst baggage images. TiP items are fictitious guns, knives and IEDs that are pseudo-randomly presented in the context of whole bags to screeners. TiP items are always of low prevalence - one published study reported on TiP detection rates using data from real screeners performing their jobs in situ [53] where the prevalence rate was set at 4%. That low prevalence rate is, however, higher than the actual incidence of these kinds of threats.

Although target prevalence remains an issue for baggage screeners, they are detecting prohibited items, including threat items, more often than might be supposed. Importantly, with the inclusion of prohibited items beyond threats, they are doing so at a higher rate than envisaged in some experimental analogues of baggage screening where target prevalence is manipulated. 

## 7. Differences in Individual Screeners

So far we have considered how studying eye movements has informed us of the challenges associated with finding and identifying prohibited items during baggage search. In doing so it seems to us that key eye movement metrics reflect different stages of processing and decision making associated with baggage search. In this final section, we consider how these metrics might be influenced by individual differences.

As a population, baggage screeners vary in their age, experience and training, in addition to their cognitive abilities and affective characteristics. Differences in screener performance are certainly contributed to by individual differences in basic perceptual processing (e.g., references [35,54]). There is evidence for reliable individual differences over time in sensitivity and some evidence of modest age-related decline that has been attributed to reducing efficiency of perceptual and cognitive abilities that cannot be overcome by years of performing the screening task [54,55].

More importantly, and perhaps not surprisingly, differences in screener performance are contributed to by training focussed on developing robust templates of prohibited items [35]. A question that emerges is whether training improves search guidance to possible prohibited items or verification for identification? While there is likely to be some improvement in both, there is good evidence that the effect of training is more striking on processes associated with verification than guidance [22,56]. The improvement in target detection that comes with training is more striking with respect to IEDs than guns, knives and other kinds of prohibited items [57]. It seems that training mostly (though not exclusively) improves the robustness of the templates that allow screeners to more readily match what is seen to what they know, and improvement is greatest when there is most to learn.

A pattern emerges of good screeners being defined as having (1) an ability to make a fast and accurate ‘clear’ decision based on the gist derived from the image; (2) excellent guidance of attention to potential prohibited items; (3) speed in verifying or rejecting item identity based on having robust target templates to match items against; while also ensuring that they are (4) sufficiently exhaustive in search to ensure all possible targets are investigated and all viable interpretations of items are tested against target templates.

While these are distinct issues that could be explored in future studies, the current literature allows only a coarser analysis, which we consider in terms of three broad categories. The first is how working memory capacity and attentional control might influence search and guidance. The second is how factors related to conservatism in decision-making influence the thoroughness of search and the time given to verify target presence and absence (and how this relates to the first category). Finally, we consider a third broad category of individual differences that may relate specifically to search through slices of a 3D data volume. 

Our goal in exploring these issues is simple. Prior evidence clearly shows that baggage screening performance is associated with basic perceptual skills and task knowledge. Beyond that, is there any evidence of systematic relationships between cognitive and affective factors and eye movement behavior when searching complex images then we might be able to use to inform the selection of baggage screeners? We ask this question in light of the four characteristics of good screening that were outlined above and in the spirit of a hypothesis worthy of exploration. We do not have a view of the relative importance of the individual differences that we discuss for baggage screening, especially since they may jointly influence the performance of any given individual.

## 8. Working Memory Capacity and Attentional Control

While working memory capacity (WMC) does not predict performance in very simple searches, it does in complex search tasks, including those that demand sustained high levels of attentional control [58,59,60]. We previously discussed how target templates are held or processed in visual working memory (e.g. references [61,62]). It follows from this that effective dual-target search will typically involve more WMC (even in searches which require long-term memory storage [63], WM is still needed for encoding, retrieving and maintaining templates [64]).

It is also conceivable that holding two targets in WM, or one target in conjunction with an item from a simultaneous memory task, may require some sort of memory organization or segregation to minimise interference. Maintaining this WM segregation may require more resources than merely searching for a single target. This is consistent with both the study noted earlier which showed that adding extra WM load can interfere with search guidance and lead to more unguided fixations [34] and with evidence that when search distractors match a color held in WM, saccades are slower and less accurate [65]. Greater WMC must therefore be advantageous in dual-target search tasks that involve the guidance of eye movements to two targets [66].

Extra resources will also help sustain high levels of attentional control and avoid erroneous eye movements due to the conflicts that must be resolved as different targets try to pull attention in different directions [67]. Furthermore, maintaining attentional control is likely to be particularly challenging when a dual-target search is coupled with low levels of target prevalence [68,69]. It is beyond the scope of the current review to discuss the relationship between WM and attention at any length (see reference [70] for more detail), but we can say that WMC predicts attentional control in an antisaccade task and, in the same study, high WMC participants exhibited less of a performance cost when switching from an anti- to a prosaccade task [71]. Similar costs may be observed when two target templates are active successively and guidance shifts from one target to another over time; not only will extra resources be needed to coordinate the switch, but residual information from previous task sets, including target templates, will also increase the likelihood of eye movements to irrelevant items [72,73,74,75]. 

As individuals search, some of the resources that could have been allocated to dual-target guidance may instead be engaged in other cognitive tasks; they may be monitoring how well they are doing in the task, or speculating on the motivation for the experiment, or making plans for the rest of the day [76]. Participants may also be motivated to hold some resources in reserve due to a built-in aversion to high levels of resource utilization that ensures resource availability for unanticipated future tasks. In summary, while it not been tested directly, it follows from these behavioral and eye movement laboratory studies that good baggage screening performance would be associated with high WMC and good attentional control.

## 9. Setting Conservative Decision Thresholds

While low target prevalence makes search and target verification more challenging, good baggage screeners will be relatively resistant to these effects; they will tend to ensure all locations that might contain a target are searched and that those locations are processed until all possible interpretations of image features are considered before deciding to clear or reject. Doing so equates to good baggage screeners setting conservative decision thresholds for terminating their inspection of individual fixations during the search and for continuing fixations before ending search, including when no target is found. The ability to set conservative decision thresholds will be influenced by training and a number of studies have examined the performance of trained screeners [55,77]. In these studies, screener performance is often characterized in terms of Signal Detection Theory measures that reflect the relationship between screeners’ hit rates and false alarm rates [78]. 

Apart from training, the setting of decision thresholds will also be influenced by the individual tendency to be ‘satisfied’ with search. ‘Satisfaction of search’ was a term used primarily in the context of radiographic screening to describe failures to detect subsequent targets following the detection of a first [79]. Different accounts of this effect exist, but it is likely that it is not related to satisfaction at all, but rather a combination of a bias towards finding subsequent targets that are similar to an initial target (perceptual set bias) and the depletion of cognitive resources associated with finding an initial target [80,81]. This phenomenon has since been renamed ‘subsequent search misses’ to reflect the contribution of these other factors [81]. Individual tendencies linked to satisfaction are an important source of individual variance in the setting of quitting thresholds and response criteria during the search [82,83,84]. Consistent with the notion of resource depletion, recent evidence links individual differences in WMC to errors of both perceptual selection (failure to fixate targets) and perceptual identification (failure of verification) during the search [85]. In a follow-up study, the effect of WMC on perceptual selection errors was attributed to quitting thresholds that were not adequately conservative; that is to say, searchers quit before they had fixated targets [68].

There are a number of psychological measures that tap into individual tendencies for satisfaction during the search. One of these is the ‘Maximization Scale’ [86], a 13-item personality scale that assesses the degree to which an individual is a ‘maximizer’, who will strive for the best outcome, or a ‘satisficer’, who will accept outcomes that are good enough. Maximization is also conceptually linked to perfectionism and it has been demonstrated that perfectionists who engage with X-ray search and object identification tasks perform these tasks more accurately and faster than other individuals [87]. Attention to detail, as assessed by a subscale of the Autism Quotient, predicts accuracy in X-ray baggage search and has been the basis of a recent scale developed specifically to assess aptitude for X-ray baggage screening, the XRIndex [88]. 

One component of trait anxiety, Intolerance of Uncertainty (IU), may also lead to a tendency to settle for poorer performance in a search. IU represents the extent to which individuals experience (and can cope with) worry about uncertain future events [89,90] and can bias decision making towards minimizing uncertainty. IU positively predict false alarm rates in a complex search task where participants searched for low prevalence color targets. In this task the color targets appeared in arrays of colored squares whose appearance changed dynamically over time through an ordered color space [6]. The increased false alarms reflect a decision to prematurely classify as targets items that are still distractors (and have yet to become targets).

Compared to simple laboratory search tasks, the complexities of X-ray search may increase the likelihood of individuals who tend to be easily satisfied, or those with greater IU, to terminate searches prematurely (i.e. lowering quitting thresholds). We suspect that there are some contingencies between the thresholds for terminating fixations both to possible targets and in target verification, WMC, maximisation and IU. At the very least, it is an area worthy of further research. 

## 10. Searching through Three-Dimensional Volumes

Earlier in this review we discussed how baggage search is merely altered, and not fundamentally changed, by advances in screening technology. One of the ways in which screening is altered by CT scanning technology is that screeners are able to search through sequential 2D slices of a 3D volume. Interacting with images in this way introduces a dynamic element into the search task, whereby the image to be searched changes dynamically as screeners move through the slices. Each 2D slice is also related to the slices either side of it in 3D space, such that moving through slices will cause objects to emerge over time in a predictable way. This technology is relatively new in baggage screening and the implications of it for the eye movements made during the search are unclear (though see reference [91] for a discussion of related issues in medical imaging ).

A priori it seems to us that searching for prohibited items with a technology that allows search to have a predictive aspect across ‘slices’ brings a risk that search can become unduly focussed on these locations. From a different paradigm, there is evidence that individual differences in WMC and IU influence the number of fixations made when monitoring dynamically changing color displays for the onset of targets [6]. For individuals with low WMC, those with high IU made fewer eye movements relative to those with low IU. This issue requires much further investigation. Nevertheless, this result emphasizes the importance of measuring eye movements as a tool to help develop our understanding some of the psychological challenges that new ways of the data visualizations bring.

## 11. General Discussion and Summary

We have known for a long time that security searches of baggage can be very difficult. Security screeners start at a disadvantage when searching through X-ray images, because those images differ in fundamental ways from real-world images. The objects do not appear in the colors that we associate with them, and overlapping objects produce a very unnatural sort of transparency. After years of research, we now have a better understanding of some of the other factors can make searches especially difficult. 

Some factors, such as the large numbers of objects in some bags and the degree to which those objects overlap one another in the image, may be addressable by developing new technology that can enhance images, perhaps by rotating them or presenting them in depth. Eye tracking has been a useful tool in assessing technologies and measuring how searchers use them, and as both the display technologies and the experimental methods advance, we can expect eye tracking studies to be even more valuable in assessing future technological advances.

Other factors that complicate X-ray security searches include limitations on cognitive mechanisms for attention and object recognition. Eye tracking data have allowed us to better understand some of these limitations, such as the prevalence effect and the dual-target cost. It may be possible to partly or fully overcome some of these limitations by developing new training methods. For instance, it may be possible to train subjects to search thoroughly even though targets rarely appear, or to guide their search effectively using multiple target templates. Once those methods are developed, additional eye tracking studies will allow us to measure their benefits.

If some of these limitations cannot be eliminated by training, it may still be possible to improve security searches by devising ways to identify those individuals with the most effective perceptual and attentional mechanisms for this type of search. The studies reviewed above suggest that testing for WMC, maximization and tolerance to uncertainty may help to find better screeners. Research on individual differences that are relevant to security search is just getting off the ground, so there is probably lots to learn here.

The eye tracking studies reviewed here have given us a better understanding of the many factors that make security searches difficult, and in some cases they are helping to find methods of improving performance. These are, of course, important contributions to the safety and well-being of large numbers of people, but there have been other benefits to emerge from this research as well. In addition to the practical improvements in detecting threats, this research has contributed to our understanding of visual search and the mental processes that control attention and object identification. For instance, one set of studies shows that decisions to stop search are not driven solely by the stimuli but are influenced by internal factors that have their origins outside of the attentional system. Another set of studies shows that subjects do not effectively guide their attention when searching for two colors if they can instead identify the target with a single shape discrimination, even though this method makes the search much less efficient. These findings open up new questions about the basic nature of attentional control that go beyond security search. We can expect that future research in this area will lead to further advances in our theoretical understanding of visual cognition, while also enhancing our abilities to detect threats.
